# Investigation of a Modified Novel Technique in Bilateral Sagittal Splitting Osteotomy Fixation: Finite Element Analysis and In Vitro Biomechanical Test

**DOI:** 10.1155/2020/8707389

**Published:** 2020-06-17

**Authors:** Li-Ren Chang, Chien-Chung Chen, Seng Feng Jeng, Yu-Ray Chen, Lain-Chyr Hwang, Ting-Sheng Lin

**Affiliations:** ^1^Department of Plastic and Reconstructive Surgery, E-Da Hospital, Kaohsiung, Taiwan; ^2^Department of Electrical Engineering, I-Shou University, Kaohsiung, Taiwan; ^3^College of Medicine, I-Shou University, Kaohsiung, Taiwan; ^4^Craniofacial Center, Department of Plastic and Reconstructive Surgery, Chang Gung Memorial Hospital, Chang Gung University, Taoyuan, Taiwan; ^5^Department of Biomedical Engineering, I-Shou University, Kaohsiung, Taiwan

## Abstract

**Purpose:**

To evaluate the biomechanical properties of the modified novel 2-hole monocortical plate fixation (2HMCPf) and traditional 4-hole monocortical plate fixation (4HMCPf) techniques in bilateral sagittal splitting osteotomy (BSSO) synthesis using a finite element analysis (FEA) and an in vitro biomechanical test with the application of a shearing loading force on a sawbone mandible model.

**Materials and Methods:**

A three-dimensional mandible models were generated using the geometry obtained from the computerized tomography image of a sawbone mandible. Plates and screws were generated and combined with the mandible in a CAD environment. The 2HMCPf and traditional 4HMCPf techniques for BSSO osteosynthesis were then analyzed under the occlusal load using the FEA. An in vitro biomechanical test was executed to verify the result of FEA. The force on fixation failure and pattern of failure were recorded.

**Results:**

The results revealed that the von Mises Stress on the mandible cortical bone (75.98 MPa) and the screw/plate (457.19 MPa) of the 2HMCPf group was lower than that of the 4HMCPf group (987.68 MPa, 1781.59 MPa). The stress concentrated on the central region of the 4HMCPf group and the distal set of the 2HMCPf group. In vitro study using the sawbone mandible model showed mechanical failure at the region of the proximal segment near the osteotomy site with the 4HMCPf group (average 32.198 N) but no failure on the fixation sites with the 2HMCPf group. Instead, the mandible sawbone fractured on the condyle neck region (average 44.953 N).

**Conclusion:**

From the biomechanical perspective, we proved that the 2HMCPf method was able to withstand a higher shearing loading force than the 4HMCPf fixation method in BSSO osteosynthesis.

## 1. Introduction

Bilateral sagittal splitting osteotomy (BSSO) with rigid internal fixation (RIF) is employed to advance the mandible in cases of retrognathism [1–3] or to set the mandible back in those of prognathism [4–6]. Rigid internal fixation (RIF) involves monocortical plate fixation with a 4-hole plate with or without central extension or bicortical screw fixation (BCSf) with 3 bicortical lag screws or positional screws placed in a triangular inverted L pattern. Despite the traditional 4-hole monocortical plate fixation (4HMCPf), a novel technique that involves using 2-hole monocortical plate fixation (2HMCPf) has been proposed [5, 7–11] in recent years. Hsu et al. evaluated the stability of 2HMCPf by assessing the amount of relapse [5]. They demonstrated that the stability of 2HMCPf was equal to that of BCSf. They also mentioned that 2HMCPf might withstand higher levels of shear stress compared to the traditional 4HMCPf [5]. However, to the best of our knowledge, there are no in vitro biomechanical studies about the resistance of shear stress with the 2HMCPf technique.

The purpose of the present study was to evaluate the biomechanical properties of 2HMCPf and the traditional 4HMCPf in BSSO osteosynthesis using the finite element analysis (FEA) and verify the result with an in vitro biomechanical test using a sawbone mandible model. Additionally, this study tried to investigate whether 2HMCPf can withstand higher stress levels than the 4HMCPf.

## 2. Materials and Methods

### 2.1. Development of a Finite Element Model of the Mandible

**Figure 1:**
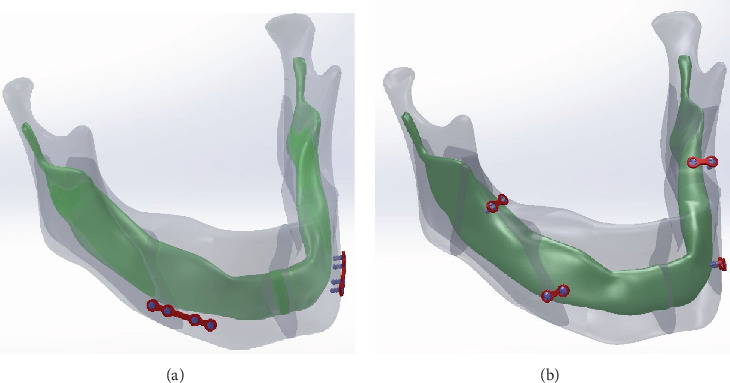
Configuration of (a) the 4HMCP (4-hole monocortical plate) and (b) the 2HMCP (2-hole monocortical plate) solid models.

The geometry of the mandible bone was obtained from the Department of Dentistry of E-Da Hospital (Kaohsiung City, Taiwan). Computed tomographic (CT) images were captured at 3 mm intervals and digitized into the DICOM format. The geometry of the miniplates and screws was obtained from the manufacturer-provided information of the commercially available product (COMPACT 2.0 MF Internal Fixation System, Synthes, USA). The three-dimensional solid models of the miniplate system and mandible bone were reconstructed and assembled using a commercial software application (SolidWorks 2008, Dassault Systèmes SolidWorks Corp., MA, USA). Because the objective of this study focused on intraoral stability, the teeth of the mandible were not incorporated into the model. The integrated model was imported to the finite element (FE) package (ANSYS 11.0, ANSYS Inc., PA, USA) and was meshed using a three-dimensional 10-node tetrahedral structural element (Figure 1).

### 2.2. Loading and Boundary Conditions

**Figure 2:**
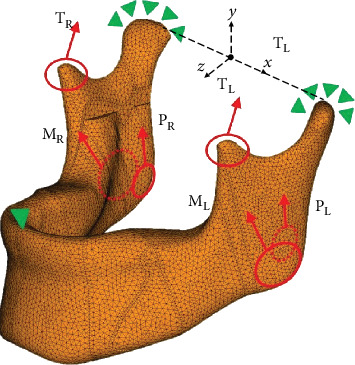
Loading and boundary conditions in the finite element analysis.

The mandible bone and miniplate system were both assumed to be composed of homogeneous, isotropic, and linear elastic materials. The mechanical properties, including the Young's modulus (*E*) and Poisson's ratio (*ν*) of the cortical bone (*E* = 13.3 GPa, *ν* = 0.224), cancellous bone (*E* = 1.33 GPa, *ν* = 0.224), miniplates, and screws (*E* = 105 GPa, *ν* = 0.33) were acquired based on previously published data [12]. The appropriate element mesh size for all the mesh models was determined to be 0.9 mm after the convergence of the FE model was calculated. Consequently, the interfaces between the bone, plate, and screws were all assumed to be bonded; however, the incision bone surfaces of both sides were set to be contacted. One node in the anterior region of the mandible was constrained in the *y*-direction in order to simulate the function of an occlusal splint that constrains the excessive motion of the mandible postoperatively (Figure 2).

After the mesh models were generated, the major muscle forces were applied, including masseter, temporalis, and pterygoid muscle forces (Table 1 and Figure 2), to simulate the occlusion force [13–15]. All the surface nodes were fixed at the mandible condyle region. Maximum von Mises stresses were evaluated in the present study to examine whether the entire construction withstood the given load applied to the mandible and plate/screw.

### 2.3. In Vitro Biomechanical Experiments

#### 2.3.1. Osteotomy

The modified Hunsuck technique and its refinement by Chang Gung craniofacial center were used for osteotomy [9, 10, 16]. The proximal osteocorticotomy site was on the lingual side of the ramus just above the lingula of mandibular. The osteotomy was extended distally to the first molar teeth, at least 5 mm behind the mental foramen on the buccal side [9, 10]. Because the aim of this study was purely on the stability of two different fixation methods on the BSSO osteosynthesis, neither advancement nor setback was performed. A 0.5 mm gap was then generated to simulate the surgical procedure involved in BSSO.

#### 2.3.2. Placement of 2HMCP and 4HMCP

**Figure 3:**
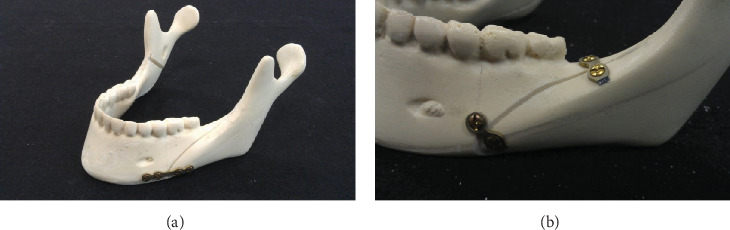
Sawbone specimen for the biomechanical test. (a) 4HMCP group; (b) 2HMCP group.

The bone plates were oriented in two different ways. The 4HMCPf group involved using a 4-hole straight plate on the distal osteotomy site with two holes on each side. The plate was placed parallel to the lower mandible border and 5 mm below the mental foramen (Figure 3(a)). The 2HMCPf group used a pair of 2-hole straight plates cut from a 4-hole straight plate. The proximal plate was placed 12 mm posterior to the second molar tooth cusp, and the distal one was placed on the osteotomy site below the first molar teeth (Figure 3(b)). The screws were inserted perpendicularly.

#### 2.3.3. In Vitro Biomechanical Experiments

Ten sawbone mandible models (Sawbone; Pacific Research Laboratories Inc., Vashon Island, WA, USA) were divided into 2HMCPf and 4HMCPf groups for the biomechanical study. Based on the study done by Ramos et al., both condyles of the sawbone mandible were fixed on the device [15]. The MTS Qtest/10 system (MTS System Co., USA) was used to apply the force onto the central incisor of the mandible to produce a shear load [17, 18]. The preload was set to 10 N at the rate of 1 mm/min. Subsequently, it was allowed to continue at the rate of 2 mm/min until the mandible fixation failed [19]. The value of the failure load and patterns of fixation failure were recorded.

## 3. Results

### 3.1. von Mises Stresses of the Mandible Bone

**Figure 4:**
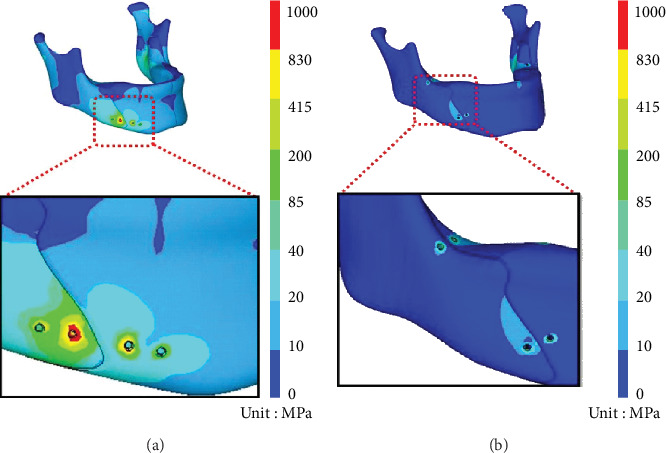
von Mises stress on the mandible bone of the (a) 4HMCP and (b) 2HMCP models.

The numerical results demonstrated that the cortical bone stresses were much greater than those of the cancellous bone; therefore, only the maximum cortical bone stress was evaluated in this study. The cortical bone stress was concentrated on the insertion sites of bone screws with the highest one close to the osteotomy site of the proximal segment, i.e., the proximal second hole (PSH) (Figure 4). The highest cortical bone stress value in the 4HMCPf group was 987.68 MPa, which was higher than the yield strength of the sawbone mandible (85 MPa). The highest cortical stress level in the 2HMCPf group was 75.98 MPa, which was lower than the sawbone mandible yield strength (85 MPa).

### 3.2. von Mises Stresses of the Plate/Screw

**Figure 5:**
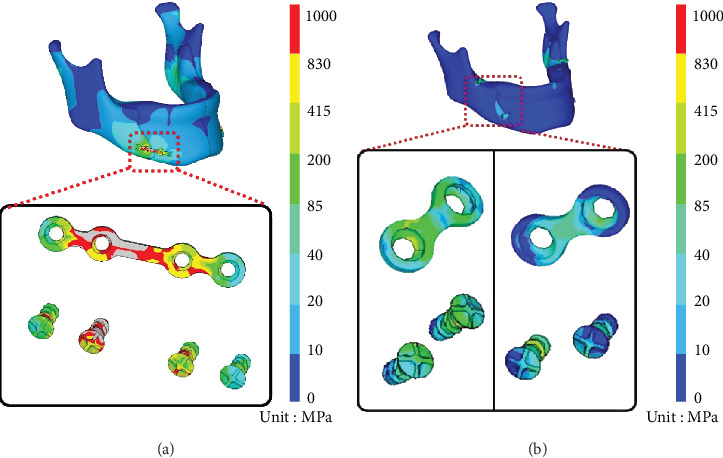
von Mises stress on the screw/plate of the (a) 4HMCP and (b) 2HMCP models.

Figure 5 presents the stress distribution of the plate/screw for each fixation group. In the 4HMCPf group, the maximum stress was 1781.59 MPa in the middle region of the plate. In the 2HMCPf group, the stress distributions concentrated on the distal 2HMCP with the maximum stress of 457.19 MPa, and lower stress of 433.53 MP on the proximal 2HMCP.

### 3.3. Results of the In Vitro Biomechanical Test

**Figure 6:**
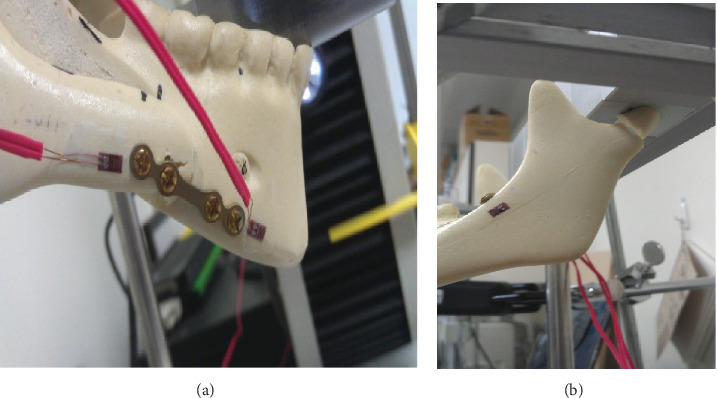
Fracture pattern in the biomechanical test. (a) Mandible bone failure and screw loosening in the 4HMCP specimen; (b) Subcondylar fracture in the 2HMCP specimen.

Figure 6 presents the results of the in vitro biomechanical test. With the 4HMCPf method, all the sawbone mandible models broke in the region of the PSH (mean failure load: 32.20 N) without plates or screws destruction. With the 2HMCPf method, all the 5 models fractured on the condyle neck (mean failure load: 42.95 N) and there was no plate and screw failures.

## 4. Discussion

### 4.1. The History and Evolution of 2HMCPf

In many studies of BSSO osteosynthesis, 4HMCPf was found to be less rigid than BCSf in response to the shearing stress produced by the masseter muscle [3, 6, 20]. In recent years, several studies have demonstrated that both BCSf and 4HMCPf resulted in equal stability in the case of setback [4, 5, 21] and advancement [2, 22] procedures in both clinical cases [4, 5] and in cadaver models [21]. However, some studies held the opposite side that BCSf is more stable than 4HMCPf [3, 20, 23]. Despite the 4HMCPf, the 2HMCPf method had been used by Professor Yu-Ray Chen for more than 15 years [10]. In 2005, Honda et al. [11] first demonstrated 2HMCPf on the postoperative X-ray after BSSO surgery. In 2009, Yu et al. [7] showed the 2HMCPf in the operative demonstration figure. In 2010, Liao et al. [8] presented their 2HMCPf cases on the postoperative X-ray images. However, the detailed 2HMCPf method had not been described in the article text until 2012, when Hsu et al. [5] first described using 3 sets of 2HMCP perpendicular to the osteotomy line over the external oblique ridge in BSSO fixation. In 2016, Sasaki et al. [9] described using 2 sets of 2HMCP for the mandible setback procedure and 3 sets of 2HMCP or 2 sets of 2HMCP with one bicortical screw for mandible advancement. In 2017, Chen et al. [10] used 2 sets of 2HMCP for the BSSO fixation and concomitant mandibular contouring. In the above studies, only Hsu et al. compared the stability of the 2HMCPf with that of BCSf [5] by measuring the relapse of the mandible position. They assumed that the 2HMCP method withstood more shear stress than the traditional 4HMCP fixation method. However, there were no formal in vitro mechanical studies to prove this. The present study proved the assumption with FEA and in vitro mechanical experiment.

### 4.2. The Reason to Use FEA First Then In Vitro Study with Synthetic Bones

The numerical results of an FEA could be credible by appropriate modeling of material properties and boundary conditions. It is beneficial not only to exclude the geometrical variances between each of the samples but also to assess the internal stress and strain distribution at the crucial region. FEA has become a popular and essential approach employed broadly in biomechanical research [24–26]. Owing to the scarcity of cadaveric samples and different properties of animal mandibles [19, 23, 27], artificial mandible bones are used [6, 28]. Using the synthetic bones can eliminate the variables associated with animal or cadaveric bone [18], minimize interspecimen variability and variations in mandibular size, shape, bone properties, and bite forced associated with sex, age, and size [14].

### 4.3. Verification of the FEA by In Vitro Test in Both Fixation Group

#### 4.3.1. The 4HMCPf Group

In the computer model used in this study, the mandible of the 4HMCPf group sustained higher von Mises stresses than that of the 2HMCP group (987.68 MPa vs. 75.98 MPa). The von Mises stresses of the plate/screw of the 4HMCPf group exhibited maximum stress in the middle region of the plate (1781.59 MPa), which was higher than the yield strength of the plate (830 MPa). This indicates that the plate possibly would break at the central part near the osteotomy site with 4HMCPf. The results of the in vitro mandible biomechanical test demonstrated that all mandible models failure occurred at the PSH, which was compatible with the numerical results of the FEA model.

#### 4.3.2. The 2HMCPf Group

The von Mises stresses of the 2HMCP revealed higher stress on the distal plate/screw (457.19 MPa) and lower stress on the proximal plate/screw (433.53 MPa). Neither of these was higher than the sawbone mandible yield strength (830 MPa). The in vitro mandible biomechanical test demonstrated no fixation failures at the fixation site but at the condyle neck.

When the force reached to average 32.198 N, the 4HMCPf group broke at the PSH. However, the 2HMCPf group broke at a higher average force of 44.953 N on the condyle neck. The fixation site remained intact without the screw loosening or the plate breaking in the 2HMCPf group. This proves that the 2HMCPf technique is better than the 4HMCPf technique to resist the shear stress. Whether the 2HMCPf is equal or closer to the stability of BCSf needs further studies to prove it.

### 4.4. Advantages and Disadvantages of Monocortical Plate Fixation

The advantages of traditional 4HMCPf are as follows: a lower degree of inferior alveolar nerve damage [29], faster recovery of inferior alveolar nerve function [30], no scars on the face from transbuccal drilling, the prevention of mandible condylar rotation [4, 31], and possible latent positional adjustability of the mandible segment at the fixation site [32]. Despite the popularity and advantages of the 4HMCP [4, 5, 21, 29, 30, 32], the disadvantages of 4HMCP fixation are as follows. First, the plate should be molded according to the shape of the mandible curve and it is not easy to fit all the holes of the plate perfectly on the mandible surface without distorting the orientation of the bone segments. Second, the 4HMCPf alone might need the “hybrid” technique that adds one additional positional screw or lag screw to strengthen the fixation [18, 31, 33, 34], no matter in mandible advancement [3] or setback [6].

The 2HMCPf method not only inherits all the advantages of 4HMCPf but also has its own ones. First, it does not require extensive bending efforts to fit a short 2-hole plate onto the mandible surface mandible. Second, the number of plates can be added in cases of advancement. Third, it sustained more shearing force, which is the most significant load that affects the stability of mandibular fixation [35]. Fourth, the 2HMCP shares the shear stress at 2 sites to resist the compressive action of the masseter muscle, which causes the clockwise rotation of the distal segment and the counterclockwise rotation of the proximal segment of the mandible [20].

### 4.5. Importance of Stable Fixation

Stable fixation is crucial to early mobilization although the functions are still not restored to a normal state [3]. The biting force right after orthognathic surgery is lower than that produced at a later date postoperatively [36, 37]. In our practice, the occlusal splint and intermaxillary fixation were removed after orthognathic surgery. Initially, the patients are unable to bite because the teeth are not yet in their appropriate or final occlusion. When the orthodontic procedure is commenced, the patients are able to swallow a soft diet. When the bone healing begins and the occlusion is restored during the orthodontic process, the patients can start chewing and consuming a solid diet. Therefore, the fixation should be strong enough to keep the bone in position and to ensure effective healing after the operation. In the long term, stability is a result of the combination of bone healing, bone inflammation, fixation rigidity, and residual muscle force exerting different vectors [3]. The 2HMCPf might be promising to meet all need.

### 4.6. Reasons to Use the Complete Mandible but Not Hemimandible Model

Tharanon demonstrated that the force on the first premolar hemimandible causes the mediolateral bending of the intermediate bar of the miniplates [21]. The molar loading mimics torsionally deforming force [17] and the incisal loading has more impact on the osteotomy site than molar loading [18]. Therefore, using the whole mandible can obviate the medial distortion force so that the shearing force can be studied specifically. The study of Ramos et al. revealed symmetrical parameters of forces acting on the whole mandible. This is crucial to the study interaction between the central loading force and symmetrical muscle forces in the present study.

### 4.7. Reasons to Use Modified Hunsuck Technique without Advancement and Setback

The 2HMCPf correlates closely to the modified Hunsuck osteotomy technique. The original Hunsuck osteotomy ends at the junction of the ramus and body of the mandible [10, 16], which results in a short proximal segment. The modification of Professor Yu-Ray Chen in 2005 extended the osteotomy to the fist molar [10]. The long proximal segment provided a longer space to place the 2HMCP. After the proximal segment and the distal segment were separated, the pterygoid protuberance on the medial mandible angle was removed for the mandible to setback. Premature contact was eliminated between the inner surface of the distal segment and the outer surface of the proximal segment so that both segments could coapt well. The 2HMCP could then be applied on the upper edge of the proximal segment. Two sets of the 2HMCP are enough for mandibular setback surgery. In mandibular advancement surgery, the long proximal segment can afford 3 sets of 2HMCP or 2sets of 2HMCP with an additional one positional screw.

Because the present study used the whole mandible, BSSO with advancement or setback would involve too many variables such as change of mandible shape, condyle position/distance, asymmetry on osteotomy sites, and different biomechanical nature of setback and advancement surgery [18, 21]. Since this is the first mechanical study of the 2HMCPf on BSSO osteosynthesis, investigation of the fixation method without mandible movement simplifies the experiment and makes a baseline for further studies.

### 4.8. Limitations

This study has a few limitations. First, there were no advancement or setback procedures. Further studies are needed. Second, we did not perform the BCS fixation technique in the destruction test. Although we can prove that 2HMCPf is more rigid than 4HMCPf to resist shear stress, further studies are needed to ascertain whether 2HMCP fixation is more effective than BCS fixation. Third, the size of the mesh in the FEA model was 0.9 mm. The smaller the size, the more precise the analysis; however, the analysis takes a longer time and needs a higher level of computer central processing unit to perform.

## 5. Conclusion

From the biomechanical perspective, the finite element analysis and an in vitro biomechanical test using a whole mandible are compatible and prove that the 2HMCPf method tolerates more shearing force than the 4HMCPf in pure BSSO osteosynthesis.

## Figures and Tables

**Table 1:** Forces of occlusion exerted by the major muscles applied onto the mandible in the finite element analysis.

Muscle	Force (*N*)		
	*x*	*y*	*z*
Right masseter (M_R_)	5.125	77.6875	8.7
Left masseter (M_L_)	-5.125	77.6875	8.7
Right temporalis (T_R_)	1.35	6.1	-7.5
Left temporalis (T_L_)	-1.35	6.1	-7.5
Right medial pterygoid (P_R_)	70.19	168.9	-38.65
Left medial pterygoid (P_L_)	-70.19	168.9	-38.65

## Data Availability

The data used to support the findings of this study are available from the corresponding author upon request.
